# Prospective comprehensive profiling of immune responses to COVID‐19 vaccination in patients on zanubrutinib therapy

**DOI:** 10.1002/jha2.639

**Published:** 2023-01-20

**Authors:** Thi H. O. Nguyen, Chhay Lim, Masa Lasica, Ashley Whitechurch, Surekha Tennakoon, Natalie R. Saunders, Lilith F. Allen, Louise C. Rowntree, Brendon Y. Chua, Lukasz Kedzierski, Hyon‐Xhi Tan, Adam K. Wheatley, Stephen J. Kent, Theo Karapanagiotidis, Suellen Nicholson, Deborah A. Williamson, Monica A. Slavin, Constantine S. Tam, Katherine Kedzierska, Benjamin W. Teh

**Affiliations:** ^1^ Department of Microbiology and Immunology University of Melbourne Parkville Victoria Australia; ^2^ Department of Infectious Diseases Peter MacCallum Cancer Centre Melbourne Victoria Australia; ^3^ Department of Haematology St Vincent's Hospital Fitzroy Victoria Australia; ^4^ Department of Clinical Haematology Peter MacCallum Cancer Centre and Royal Melbourne Hospital Melbourne Victoria Australia; ^5^ ARC Centre of Excellence in Convergent Bio‐Nano Science and Technology University of Melbourne Melbourne Victoria Australia; ^6^ Melbourne Sexual Health Centre, Infectious Diseases Department, Alfred Health, Central Clinical School Monash University Melbourne Victoria Australia; ^7^ Victorian Infectious Diseases Reference Laboratory The Peter Doherty Institute for Infection and Immunity Melbourne Victoria Australia; ^8^ Walter and Eliza Hall Institute of Medical Research Parkville Victoria Australia; ^9^ Department of Infectious Diseases University of Melbourne, The Peter Doherty Institute for Infection and Immunity Melbourne Victoria Australia; ^10^ Sir Peter MacCallum Department of Oncology University of Melbourne Parkville Victoria Australia; ^11^ Department of Haematology, Alfred Hospital Monash University Melbourne Victoria Australia

**Keywords:** response, SARS‐CoV‐2, vaccination, zanubrutinib

## Abstract

Zanubrutinib‐treated and treatment‐naïve patients with chronic lymphocytic leukaemia (CLL) or Waldenstrom's macroglobulinaemia were recruited in this prospective study to comprehensively profile humoral and cellular immune responses to COVID‐19 vaccination. Overall, 45 patients (median 72 years old) were recruited; the majority were male (71%), had CLL (76%) and were on zanubrutinib (78%). Seroconversion rates were 65% and 77% following two and three doses, respectively. CD4^+^ and CD8^+^ T‐cell response rates increased with third dose. In zanubrutinib‐treated patients, 86% developed either a humoral or cellular response. Patients on zanubrutinib developed substantial immune responses following two COVID‐19 vaccine doses, which further improved following a third dose.

## 1 INTRODUCTION

Vaccination can be an effective measure to prevent infection with SARS‐CoV‐2, but specific treatments such as Bruton's tyrosine kinase (BTK) inhibitors are known to negatively impact humoral vaccine responses [1]. In early data, rates of seropositivity following one and two doses of COVID‐19 vaccine were lowest in chronic lymphocytic leukaemia (CLL) patients and in patients treated with BTK inhibitors [1]. Data are now emerging on humoral responses in CLL patients after the use of a third vaccine dose as well as the importance of early cellular, predominantly T‐cell responses [2, 3, 4].

Zanubrutinib is a next‐generation BTK inhibitor, which is more selective and has potentially less off‐target activity, but its impact on the range of immune responses to COVID‐19 vaccination has not been studied in detail [5]. As such, this prospective study was conducted to evaluate a comprehensive range of humoral and cellular immune responses to three doses of COVID‐19 vaccine and the use of heterologous vaccination schedule in a well‐defined group of patients with CLL and Waldenstrom's macroglobulinaemia (WM) treated with zanubrutinib.

## 2 METHODS

A multicentre prospective observational cohort study was conducted from March 2021 to March 2022 in Melbourne, Victoria, Australia. Patients with CLL and WM currently receiving zanubrutinib therapy and treatment‐naïve patients (control cohort) were recruited during this period. Vaccination was administered as standard of care in line with the Australian vaccination program; ChAdOx1 and BNT162b2 vaccines were available to participants at the start of the study period. For the third dose, mRNA vaccines were recommended. Blood samples were collected at baseline prior to vaccination, 21–30 days following first and second doses and 6 months following the second dose, which also encompasses the third dose (Figure S1).

All available blood samples at each time point were analysed for antibodies against anti‐receptor‐binding domain (RBD), neutralising antibodies (nAb) against SARS‐CoV‐2 and SARS‐CoV‐2‐specific B cells and CD4^+^ and CD8^+^ T cells according to methods summarised in Supplementary Table S1 [6–8]. All samples were also tested for SARS‐CoV‐2 nucleocapsid antibody for previous COVID‐19 infection. In brief, the following definitions were utilised to classify immune responses: seroconversion was a fourfold rise in RBD antibodies from baseline, nAb response by achievement of >20% inhibition by surrogate virus neutralisation test and B‐cell response by an increase in percentage of SARS‐CoV‐2‐specific B cells. CD4^+^ and CD8^+^ T‐cell responses were defined by a twofold increase in SARS‐CoV‐2‐specific CD4^+^ and CD8^+^ T cells, measured by activation‐induced marker assay for CD134^+^CD137^+^CD4^+^ and CD69^+^CD137^+^CD8^+^ T cells (Table S1).

Clinical characteristics and immune responses were compared between treatment‐naïve and zanubrutinib‐treated patients, underlying disease and primary two‐dose vaccine type utilising Fisher's exact, chi‐square and Mann–Whitney analysis with *p* < 0.05 considered statistically significant. Analyses were performed using Prism version 9.4.1 (GraphPad software, CA, USA). This study was approved by the Human Research Ethics Committee, Peter MacCallum Cancer Centre.

## 3 RESULTS

Forty‐five patients were recruited and CLL (34/45, 76%) was the most common underlying disease, with the remaining patients diagnosed with WM (Table 1). Patients had a median age of 72 years (interquartile range [IQR] 66–79 years), and the majority of them were male (71%). Thirty‐five (78%) patients were receiving treatment with zanubrutinib, while 10 (22%) were treatment naïve. Most patients were on zanubrutinib as the first‐line therapy (19/35, 54%).

**TABLE 1:** Patient characteristics and vaccination details

Demographics	*N* = 45 (%)
Age (IQR)	72 years (66–79 years)
Sex	
Male	32 (71%)
Female	13 (29%)
Underlying malignancy	
CLL	34 (76%)
WM	11 (24%)
Treatment	
Zanubrutinib^a^	35 (78%)
Treatment naïve	10 (22%)
Number of lines of therapy, median (IQR) (zanubrutinib group)	1 (1–2)
Zanubrutinib‐treated group (*n* = 35)	
First‐line therapy	19 (54%)
Second‐line of therapy	10 (29%)
Third or greater line of therapy	6 (17%)
Anti‐CD20 antibody therapy in the last 12 months	0 (0%)
Number of vaccine doses	
Two doses	45 (100%)
Three doses	42 (93%)
Time between second and third dose, months (IQR)	4 (3–5 months)
Time between third dose and third time point, months (IQR)	2 (1–3 months)
Vaccine type for initial two doses	
ChAdOx1	41 (91%)
BNT162b2	4 (9%)
Type of three‐dose schedule (*n* = 42)	
Heterologous^b^	35 (83%)
Homologous^c^	7 (17%)

Abbreviations: CLL, chronic lymphocytic leukaemia; IQR, interquartile range; WM, Waldenstrom's macroglobulinaemia.

^a^ CLL (*n* = 25); WM (*n* = 10).

^b^ Heterologous: ChAdOx1–ChAdOx1–BNT162b2 (*n* = 31), ChAdOx1–ChAdOx1–mRNA1273 (*n* = 4). Treatment naïve (*n* = 6), CLL‐zanubrutinib (*n* = 20), WM‐zanubrutinib (*n* = 9).

^c^ Homologous: BNT162b2 × 3 (*n* = 3), ChAdOx1 × 3 (*n* = 3), BNT162b2–BNT162b2–mRNA1273 (*n* = 1). Treatment naïve (*n* = 2), CLL‐zanubrutinib (*n* = 4), WM‐zanubrutinib (*n* = 1).

The majority of patients received ChAdOx1 (41/45, 91%) as their initial two‐dose schedule, while the remaining received BNT162b2 vaccine. All patients received two doses, while 42 (93%) had three doses, with a median time of 4 months (IQR 3–5) from the second to third dose. The BNT162b2 vaccine was most commonly used for the third dose (34/42, 81%). Eighty‐three percent of patients had a heterologous vaccination schedule (different vaccine platform for the third vaccine dose). At baseline, no patients had prior COVID‐19 infection, while two patients developed COVID‐19 during the study period and were excluded from subsequent analysis (Figure S1). These infections were mild and managed as outpatients.

Following two and three doses of the COVID‐19 vaccine, 65% and 77% of all participants achieved seroconversion, respectively. In terms of nAb response, 42% and 64% of patients mounted an nAb response against wild‐type SARS‐CoV‐2 after two and three doses, respectively. The proportion of patients achieving SARS‐CoV‐2‐specific B‐cell responses, CD4^+^ and CD8^+^ responses increased from 4% to 36% (*p* = 0.02), 69% to 85% and 63% to 69%, respectively, with the third dose.

Humoral and cellular immune response rates were lower in zanubrutinib‐treated compared to treatment‐naïve patients following two and three doses, with detailed results in Table 2. In particular, nAb response rates were significantly lower (two doses, 26% vs. 86%, *p* = 0.02). Mean RBD antibody titres, nAb percentage inhibition and SARS‐CoV‐2‐specific B cells were significantly lower following second and third doses in zanubrutinib‐treated compared to treatment‐naïve patients (Figure S2). The mean CD4^+^ and CD8^+^ T‐cell counts by treatment status are also summarised in Figure S2.

**TABLE 2:** Overall immune response rates following two and three doses of COVID‐19 vaccine by treatment status

Immune response	After two doses				After three doses				Two versus three doses
Treatment status	Treatment naive	Zanubrutinib	TN versus ZB, *p*	Overall	Treatment naive	Zanubrutinib	TN versus ZB, *p*	Overall	Overall *p*
Seroconversion (RBD antibody)	88% (7/8)	57% (13/23)	0.20	65% (20/31)	100% (5/5)	71% (12/17)	0.29	77% (17/22)	0.38
Neutralising antibody (wild type)	86% (6/7)	26% (5/19)	0.02	42% (11/26)	100% (5/5)	53% (9/17)	0.12	64% (14/22)	0.16
SARS‐CoV‐2 B cell	13% (1/8)	0% (0/20)	0.29	4% (1/28)	100% (3/3)	13% (1/8)	0.02	36% (4/11)	0.02
SARS‐CoV‐2 CD4^+^ T cells	56% (5/9)	71% (17/23)	0.38	69% (22/32)	75% (3/4)	89% (8/9)	>0.99	85% (11/13)	0.46
SARS‐CoV‐2 CD8^+^ T cells	56% (5/9)	65% (15/23)	0.70	63% (20/32)	50% (2/4)	78% (7/9)	0.53	69% (9/13)	0.74

Abbreviations: RBD, receptor‐binding domain; TN, treatment naïve; ZB, zanubrutinib.

In zanubrutinib patients who did not achieve an RBD antibody response after two doses, 70% (7/10) and 50% (5/10) achieved SARS‐CoV‐2‐specific CD4^+^ and CD8^+^ T‐cell responses, respectively. Overall, 86% (21/24) of zanubrutinib‐treated patients achieved either a humoral or cellular response after two doses, while 38% (9/24) achieved both a cellular and humoral response. Of 10 patients who did not achieve an RBD‐serologic response after two doses, only one patient (10%) seroconverted after the third dose. After three doses, 81% (13/16) of zanubrutinib‐treated patients maintained a positive humoral or T‐cell response. There were no differences in immune responses according to underlying disease (CLL vs. WM) (Table S2).

Seroconversion, CD4^+^ and CD8^+^ T‐cell response rates were higher by 7%–14% in patients who received BNT162b2 as their initial two doses compared to ChAdOx1, but the differences were not statistically significant. The RBD antibody seroconversion rate and CD4^+^ and CD8^+^ T‐cell responses were higher following heterologous three‐dose vaccination compared to homologous vaccination schedule, but the differences did not reach statistical significance (Table S3).

## 4 DISCUSSION

Compared to published literature, the design of our study had three advantageous aspects: (1) patients were enrolled prospectively and tested uniformly; (2) we used a fourfold rise in antibody levels to define serological response, in line with established definitions utilised in influenza vaccination studies [9]; and (3) T‐cell responses were assessed at all time points. These differences may explain why a higher proportion of zanubrutinib‐treated patients mounted an antibody response (57% for RBD, 26% for nAb) compared to the studies of other BTK inhibitor and B‐cell targeting therapies (∼35% seropositivity rate) [1]. The response rate was observed despite the absence of a SARS‐CoV‐2‐specific B‐cell response. Other explanations include the frequent use of zanubrutinib as first‐line therapy and the lack of recent anti‐CD20 antibody therapy. However, response rates are still lower compared to treatment‐naïve patients, supporting the recommendation for vaccination prior to commencement of targeted therapies [1, 3]. Immune responses were similar between CLL and WM patients, but potential differences may not have been detected due to limited patient numbers.

SARS‐CoV‐2 T‐cell responses, in particular CD8^+^ T‐cell responses, may be critical for protection against severe COVID‐19 disease, especially for patients with haematological malignancies [10, 11]. While on zanubrutinib, the proportion of patients who developed CD4^+^ and CD8^+^ T‐cell responses was 7%–18% higher than the proportion of patients who developed RBD antibody responses. Response rates of 70% achieved with two doses and 80%–90% with three doses were comparable to rates in CLL patients not on active therapy and higher than patients with prior lines of therapy or on ibrutinib [2, 12, 13]. Higher BTK selectivity and lower off‐target activity of zanubrutinib on other kinases, such as interleukin‐2‐inducible T‐cell kinase compared to ibrutinib may contribute to improved T‐cell responses [14]. In patients without an RBD antibody response after two doses, 70% mounted a CD4^+^ and 50% a CD8^+^ T‐cell response. In aggregate, 86% of patients on zanubrutinib had either a humoral and/or cellular response, supporting the benefit of vaccinating such patients.

Following the third dose, higher proportion of patients achieved humoral and cellular responses, supporting the use of three COVID‐19 vaccine doses in patients on BTK inhibitors. In contrast to other studies, 90% of patients in our study received an adenovirus vectored vaccine for the initial two doses, while the majority (83%) received a heterologous three‐dose vaccination [1, 3]. The use of heterologous vaccination has been suggested as an approach to improve vaccine responses [15]. In this study, a higher frequency of patients achieved humoral and cellular responses with heterologous three‐dose vaccination compared to homologous vaccination, but with limited patient numbers, the differences were not statistically significant. Further larger studies are required to evaluate this strategy.

This study has several limitations, namely, the homogenous disease treatment, relatively small control patient cohort, the different approaches to T‐cell response and definition of RBD antibody response may limit comparability with other studies. However, our study uniquely evaluated a comprehensive range of humoral and cellular responses following three doses of COVID‐19 vaccine in patients treated with the latest generation BTK inhibitor.

Zanubrutinib‐treated patients achieved substantial serological and cellular immune responses following vaccination with two doses of COVID‐19 vaccine, and response rates further improved with the use of a third dose. The use of heterologous vaccination schedules requires further study.

## AUTHOR CONTRIBUTIONS

Monica A. Slavin, Constantine S. Tam, Katherine Kedzierska, Benjamin W. Teh and Thi H.O. Nguyen designed the study. Chhay Lim, Masa Lasica, Ashley Whitechurch, Constantine S. Tam and Benjamin W. Teh recruited patients for this study. Thi H.O. Nguyen, Surekha Tennakoon, Natalie R. Saunders, Lilith F. Allen, Louise C. Rowntree, Brendon Y. Chua, Lukasz Kedzierski, Hyon‐Xhi Tan, Adam K. Wheatley, Stephen J. Kent, Theo Karapanagiotidis, Suellen Nicholson and Deborah A. Williamson performed laboratory analysis and experiments. Thi H.O. Nguyen, Chhay Lim, Lilith F. Allen, Louise C. Rowntree and Benjamin W. Teh analysed results and prepared tables and figures. Benjamin W. Teh, Monica A. Slavin, Constantine S. Tam, Katherine Kedzierska and Thi H.O. Nguyen wrote the paper with input from all authors.

## CONFLICTS OF INTEREST

Benjamin W. Teh has received research funding from MSD, Sanofi and Seqirus and is on the advisory board for Moderna, CSL‐Behring and Takeda. All other authors declare they have no relevant conflicts of interest.

## ETHICS STATEMENT

This study was approved by the Human Research Ethics Committee, Peter MacCallum Cancer Centre (HREC/74260/PMCC‐2021).

## PATIENT CONSENT STATEMENT

All participants provided written informed consent.

## Supporting information

Supporting Information

Supporting Information

Supporting Information

Supporting Information

## Data Availability

For the study protocol and de‐identified pooled data for an human research ethics committee approved study, please contact corresponding author. Individual patient data will not be shared and data transfer agreement will be required.
