# Supplementary figures and images for: Prospective comprehensive profiling of immune responses to COVID‐19 vaccination in patients on zanubrutinib therapy
Source: EJHaem. 2023 Jan 20;4(1):216–20. doi: 10.1002/jha2.639 (PMC9928803; doi:10.1002/jha2.639)

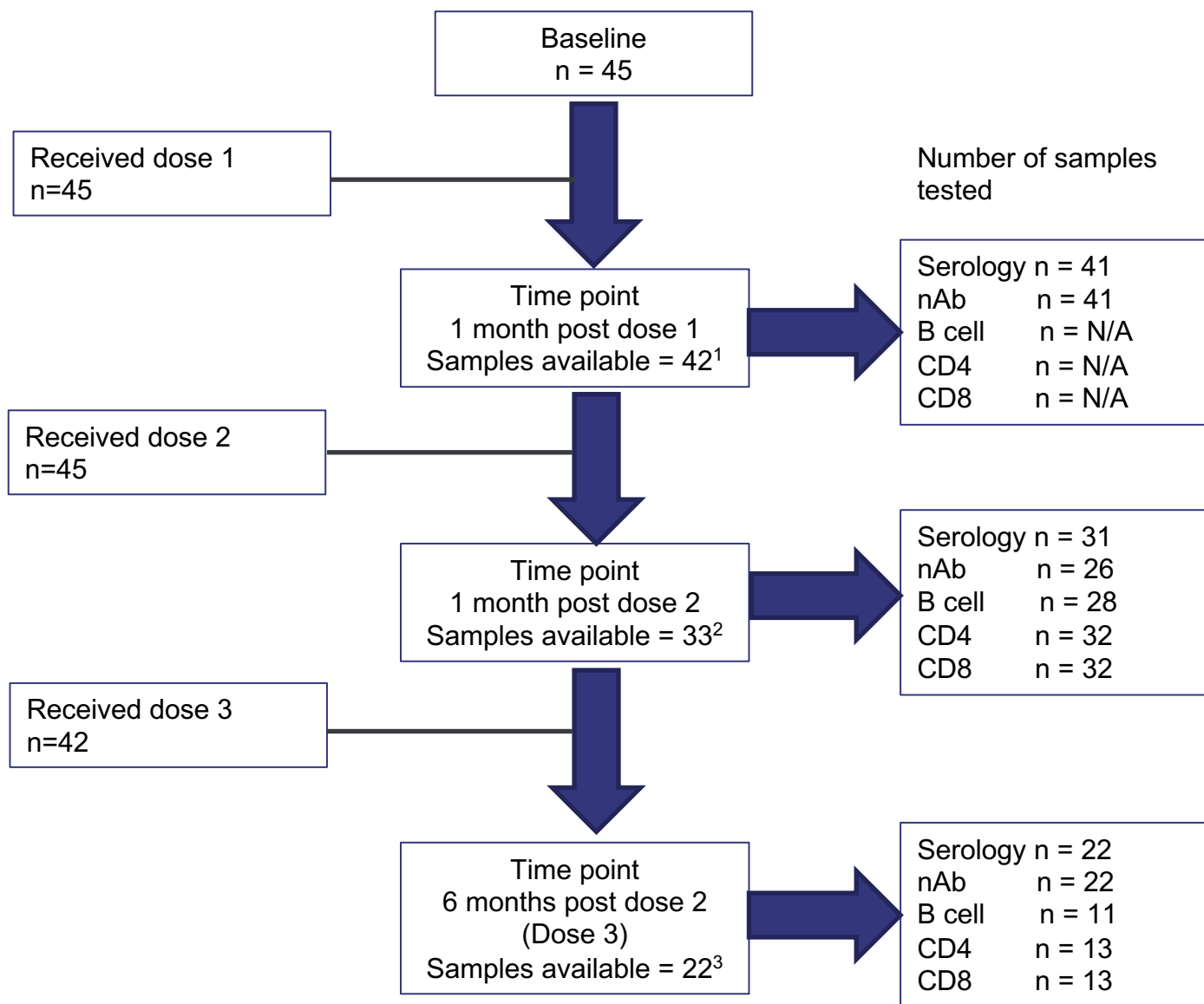

Supplementary Figure 1

Supplement: Supplementary file 1 — Supporting Information [file JHA2-4-216-s002.pdf]

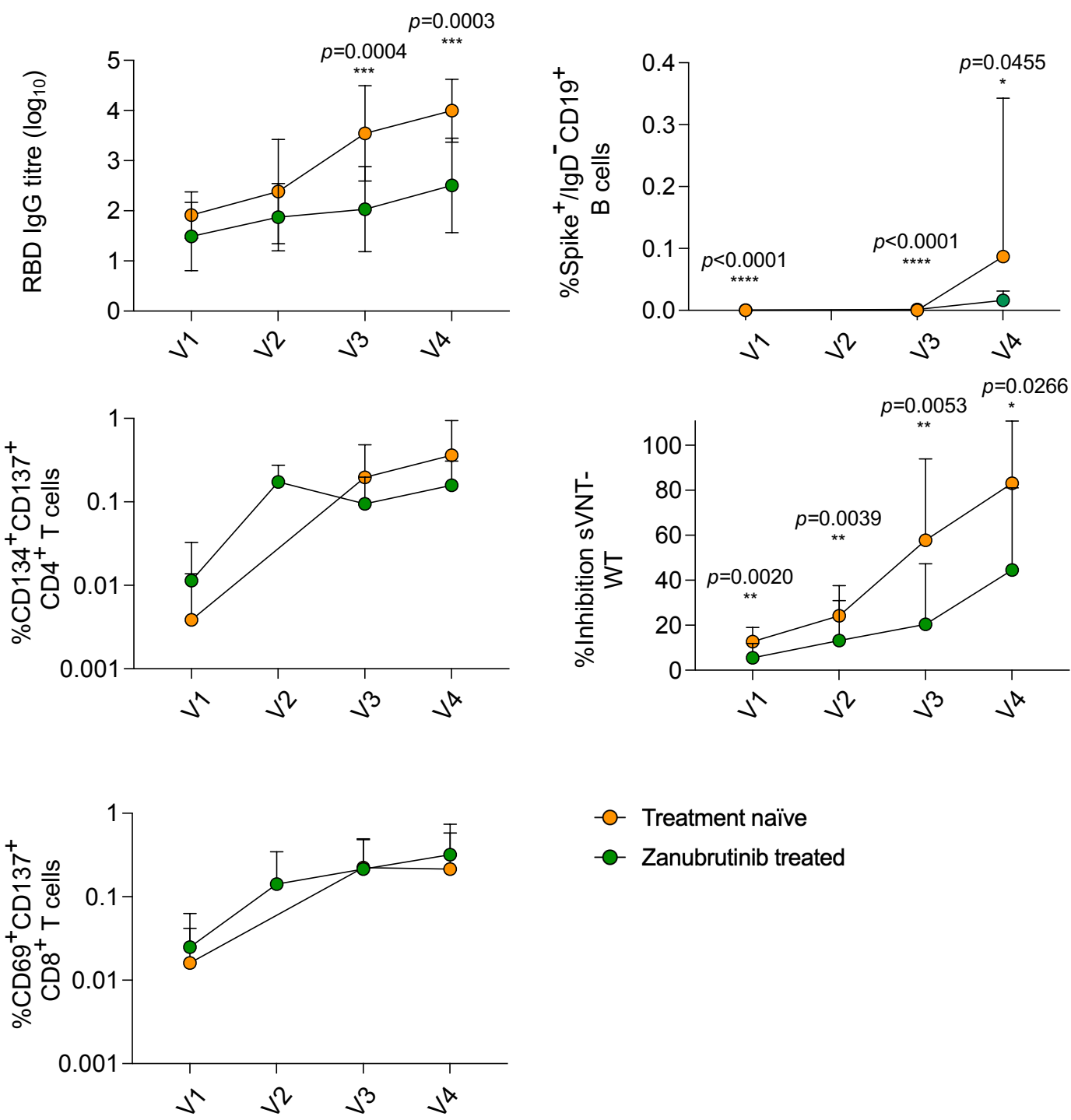

Supplementary Figure 2

Supplement: Supplementary file 2 — Supporting Information [file JHA2-4-216-s001.pdf]
