# Supplementary material for: Prospective comprehensive profiling of immune responses to COVID‐19 vaccination in patients on zanubrutinib therapy
Source: EJHaem. 2023 Jan 20;4(1):216–20. doi: 10.1002/jha2.639 (PMC9928803; doi:10.1002/jha2.639)
Supplement: Supplementary file 3 — Supporting Information [file JHA2-4-216-s004.docx]

**Supplementary Figure Legend**

**Supplementary Figure 1. Patient and sample flow during the study period**

^1^ 3 samples not available (unwell = 2, lost to follow up = 1)

^2^ 1 sample excluded (due to SARS-CoV-2 infection),11 samples not available (unwell = 2, lost to follow up = 9)

^3^ 1 samples excluded (due to SARS-CoV-2 infection), 3 samples excluded (no third dose), 19 samples not available (withdraw = 1, lost to follow up = 18)

**Supplementary Figure 2. Levels of SARS-CoV-2 specific antibody titre and cell counts over time, by treatment status**

V1 = baseline, V2 = sampling at ~1 month post dose 1, V3 = sampling at ~1 month post dose 2, V4 = sampling at ~6 months post dose 2

Dots and cross-hairs represent mean and standard deviation

RBD; receptor binding domain; sVNT: surrogate virus neutralisation test; WT: wild type
