# Supplementary material for: Prospective comprehensive profiling of immune responses to COVID‐19 vaccination in patients on zanubrutinib therapy
Source: EJHaem. 2023 Jan 20;4(1):216–20. doi: 10.1002/jha2.639 (PMC9928803; doi:10.1002/jha2.639)
Supplement: Supplementary file 4 — Supporting Information [file JHA2-4-216-s003.docx]

| **Immune markers** | **Methods** | **Reference** | **Definition of positive response** |
| --- | --- | --- | --- |
| SARS-CoV-2 spike specific antibody | In-house ELISA, measurement of RBD antibody | (Nguyen*, et al* 2021) | Seroconversion = 4-fold rise in antibody titres |
| SARS-CoV-2 nucleocapsid antibody | ECLIA, Nucleocapsid antibody (IgG) | Elecsys Anti-SARS-CoV-2 kit on Roche e601 analyser  Roche, Switzerland | Cut off index  ≥ 1.0 = reactive |
| Neutralising antibody | Surrogate virus neutralization test  ELISA assay, inhibition of RBD and human angiotensin converting enzyme-2 binding | (Zhang*, et al* 2022) | Inhibition of binding ≥20% to wild-type strain |
| SARS-CoV-2-specific B cells | PBMCs incubated with recombinant spike fluorescent probes for measuring memory spike-specific B-cells | (Nguyen*, et al* 2021) | Detection of increase in SARS-CoV-2-specific B cell frequency |
| SARS-CoV-2-specific T cells | CD4^+^/CD8^+^ T cells incubated overnight with overlapping peptides of SARS-CoV-2 spike protein followed by followed by AIM assay to measure CD134^+^CD137^+^CD4^+^ and CD69^+^CD137^+^CD8^+^ T cells | (Juno*, et al* 2020) | 2-fold increase in SARS-CoV-2 specific CD4^+^/CD8^+^ T cell frequency |

ELISA:enzyme-linked immunosorbent assay; RBD: receptor binding domain; ECLIA: electrochemiluminescence immunoassay; PBMC: peripheral blood mononuclear cells; AIM: activation immune marker

**Supplementary Table 1: Summary of immune markers, analysis methods and definitions of positive response**

| Immune response | After 2-doses | | | | After 3-doses | | | |
| --- | --- | --- | --- | --- | --- | --- | --- | --- |
| Treatment status | CLL | WM | p-value | Overall | CLL | WM | p-value | Overall |
| Seroconversion  (RBD antibody) | 63% (15/24) | 71% (5/7) | >0.99 | 65% (20/31) | 69% (11/16) | 100% (6/6) | 0.27 | 77% (17/22) |
| Neutralising antibody  (wild type) | 35% (7/20) | 67% (4/6) | 0.35 | 42% (11/26) | 56% (9/16) | 83% (5/6) | 0.35 | 64% (14/22) |
| SARS-CoV-2  B cell | 5% (1/22) | 0%  (0/6) | >0.99 | 4% (1/28) | 43% (3/7) | 25% (1/4) | >0.99 | 36% (4/11) |
| SARS-CoV-2  CD4^+^ | 72% (18/25) | 57% (4/7) | 0.65 | 69% (22/32) | 78% (7/9) | 100% (4/4) | >0.99 | 85% (11/13) |
| SARS-CoV-2  CD8^+^ | 68% (17/25) | 43% (3/7) | 0.38 | 63% (20/32) | 67% (6/9) | 75% (3/4) | >0.99 | 69% (9/13) |

CLL: chronic lymphocytic leukaemia; WM: Waldenstrom’s macroglobulinaemia; RBD: receptor binding domain

**Supplementary Table 2: Overall immune response rates following 2 and 3 doses of COVID-19 vaccine by underlying disease**

| **Immune response** | **After 2-doses** | | | | **After 3-doses** | | | |
| --- | --- | --- | --- | --- | --- | --- | --- | --- |
| Vaccination schedule | BNT162b2 | ChAdOx1 | *P* | Overall | Heterologous | Homologous | *P* | Overall |
| Seroconversion  (RBD antibody) | 75% (3/4) | 63% (17/27) | >0.99 | 65% (20/31) | 78% (14/18) | 75% (3/4) | >0.99 | 77% (17/22) |
| Neutralising antibody  (Wild type) | 75% (3/4) | 36% (8/22) | 0.28 | 42% (11/26) | 67% (12/18) | 50% (2/4) | 0.60 | 64% (14/22) |
| SARS-CoV-2  B-cells | 0% (0/3) | 4% (1/25) | >0.99 | 4% (1/28) | 30% (3/10) | 100% (1/1) | 0.36 | 36% (4/11) |
| SARS-CoV-2  CD4^+^ T-cells | 75% (3/4) | 68% (19/28) | >0.99 | 69% (22/32) | 90% (9/10) | 67% (2/3) | 0.42 | 85% (11/13) |
| SARS-CoV-2  CD8^+^ T-cells | 75% (3/4) | 61% (17/28) | >0.99 | 63% (20/32) | 70% (7/10) | 67% (2/3) | >0.99 | 69% (9/13) |

**Supplementary Table 3. Overall immune response rates following 2 and 3 doses of COVID-19 vaccine by vaccine type and schedule**

**References**

Juno, J.A., Tan, H.X., Lee, W.S., Reynaldi, A., Kelly, H.G., Wragg, K., Esterbauer, R., Kent, H.E., Batten, C.J., Mordant, F.L., Gherardin, N.A., Pymm, P., Dietrich, M.H., Scott, N.E., Tham, W.H., Godfrey, D.I., Subbarao, K., Davenport, M.P., Kent, S.J. & Wheatley, A.K. (2020) Humoral and circulating follicular helper T cell responses in recovered patients with COVID-19. *Nat Med,* **26,** 1428-1434.

Nguyen, T.H.O., Rowntree, L.C., Petersen, J., Chua, B.Y., Hensen, L., Kedzierski, L., van de Sandt, C.E., Chaurasia, P., Tan, H.X., Habel, J.R., Zhang, W., Allen, L.F., Earnest, L., Mak, K.Y., Juno, J.A., Wragg, K., Mordant, F.L., Amanat, F., Krammer, F., Mifsud, N.A., Doolan, D.L., Flanagan, K.L., Sonda, S., Kaur, J., Wakim, L.M., Westall, G.P., James, F., Mouhtouris, E., Gordon, C.L., Holmes, N.E., Smibert, O.C., Trubiano, J.A., Cheng, A.C., Harcourt, P., Clifton, P., Crawford, J.C., Thomas, P.G., Wheatley, A.K., Kent, S.J., Rossjohn, J., Torresi, J. & Kedzierska, K. (2021) CD8(+) T cells specific for an immunodominant SARS-CoV-2 nucleocapsid epitope display high naive precursor frequency and TCR promiscuity. *Immunity,* **54,** 1066-1082 e1065.

Zhang, W., Chua, B.Y., Selva, K.J., Kedzierski, L., Ashhurst, T.M., Haycroft, E.R., Shoffner-Beck, S.K., Hensen, L., Boyd, D.F., James, F., Mouhtouris, E., Kwong, J.C., Chua, K.Y.L., Drewett, G., Copaescu, A., Dobson, J.E., Rowntree, L.C., Habel, J.R., Allen, L.F., Koay, H.F., Neil, J.A., Gartner, M.J., Lee, C.Y., Andersson, P., Khan, S.F., Blakeway, L., Wisniewski, J., McMahon, J.H., Vine, E.E., Cunningham, A.L., Audsley, J., Thevarajan, I., Seemann, T., Sherry, N.L., Amanat, F., Krammer, F., Londrigan, S.L., Wakim, L.M., King, N.J.C., Godfrey, D.I., Mackay, L.K., Thomas, P.G., Nicholson, S., Arnold, K.B., Chung, A.W., Holmes, N.E., Smibert, O.C., Trubiano, J.A., Gordon, C.L., Nguyen, T.H.O. & Kedzierska, K. (2022) SARS-CoV-2 infection results in immune responses in the respiratory tract and peripheral blood that suggest mechanisms of disease severity. *Nat Commun,* **13,** 2774.
